# Widespread occurrence of chitinase-encoding genes suggests the *Endozoicomonadaceae* family as a key player in chitin processing in the marine benthos

**DOI:** 10.1038/s43705-023-00316-7

**Published:** 2023-10-14

**Authors:** Daniela M. G. da Silva, Filipa R. Pedrosa, M. Ângela Taipa, Rodrigo Costa, Tina Keller-Costa

**Affiliations:** 1 Department of Bioengineering, Instituto Superior Técnico, University of Lisbon, Av. Rovisco Pais, 1049−001 Lisbon, Portugal; 2 iBB—Institute for Bioengineering and Biosciences and i4HB—Institute for Health and Bioeconomy, Instituto Superior Técnico, Av. Rovisco Pais, 1049−001 Lisbon, Portugal

**Keywords:** Microbial ecology, Symbiosis, Water microbiology

## Abstract

Chitin is the most abundant natural polymer in the oceans, where it is primarily recycled by chitin-degrading microorganisms. *Endozoicomonadaceae* (*Oceanospirillales*) bacteria are prominent symbionts of sessile marine animals, particularly corals, and presumably contribute to nutrient cycling in their hosts. To reveal the chitinolytic potential of this iconic, animal-dwelling bacterial family, we examined 42 publicly available genomes of cultured and uncultured *Endozoicomonadaceae* strains for the presence of chitinase-encoding genes. Thirty-two of 42 *Endozoicomonadaceae* genomes harbored endo-chitinase- (EC 3.2.1.14), 25 had exo-chitinase- (EC 3.2.1.52) and 23 polysaccharide deacetylase-encoding genes. Chitinases were present in cultured and uncultured *Endozoicomonadaceae* lineages associated with diverse marine animals, including the three formally described genera *Endozoicomonas*, *Paraendozoicomonas* and *Kistimonas*, the new genus *Candidatus* Gorgonimonas, and other, yet unclassified, groups of the family. Most endo-chitinases belonged to the glycoside hydrolase family GH18 but five GH19 endo-chitinases were also present. Many endo-chitinases harbored an active site and a signal peptide domain, indicating the enzymes are likely functional and exported to the extracellular environment where endo-chitinases usually act. Phylogenetic analysis revealed clade-specific diversification of endo-chitinases across the family. The presence of multiple, distinct endo-chitinases on the genomes of several *Endozoicomonadaceae* species hints at functional variation to secure effective chitin processing in diverse micro-niches and changing environmental conditions. We demonstrate that endo-chitinases and other genes involved in chitin degradation are widespread in the *Endozoicomonadaceae* family and posit that these symbionts play important roles in chitin turnover in filter- and suspension-feeding animals and in benthic, marine ecosystems at large.

## Introduction

Chitin, the unbranched polymer of β−1,4-N-acetylglucosamine, is the second-most abundant natural polysaccharide on earth after cellulose, with global production rates estimated at 10^12^–10^14^ tonnes per year [1, 2]. It is a major constituent of fungal cell walls, marine diatoms, crustaceans, and zooplankton, and difficult to decompose in nature [1, 3]. The water insolubility of chitin contributes to the formation of ‘marine snow’ in marine ecosystems [4]. Nevertheless, chitin particles rarely accumulate on the seafloor as they are degraded by bacteria which use chitin as carbon and nitrogen sources [5]. Filter- and suspension-feeding animals, such as sponges and corals, likely process numerous chitin-rich particles present in the water column and may thus host a microbiome well adapted to chitinous food processing [6].

The gammaproteobacterial family *Endozoicomonadaceae*, first described in 2018, has a worldwide distribution and lives associated with marine eukaryotic organisms, including corals, sponges, mollusks, bryozoans, ascidians, and fishes [7]. This family currently comprises three validly published genera: *Endozoicomonas, Parendozoicomonas* and *Kistimonas* [7]. Based on metagenome-assembled-genome (MAGs) data, we recently proposed the novel genus *Candidatus* Gorgonimonas along with two candidate species, Gorgonimonas eunicellae and Gorgonimonas leptogorgiae, found to be specifically associated with temperate octocorals (Octocorallia) [8]. *Endozoicomonadaceae* symbionts can represent over 90% of a coral bacterial community [9] and are presumed indicators of coral health [9, 10]. Owing to their abundance in corals and ubiquity in marine ecosystems, the roles played by *Endozoicomonadaceae* symbionts of marine animals are of increasing research interest yet their precise functions across multiple symbioses are still poorly understood. A major limitation is the small number of cultured representatives available [11] and the apparent difficulty to manipulate these symbionts in the laboratory. Genome analyses suggested that *Endozoicomonadaceae* symbionts participate in amino acid and B vitamin supply [8, 12–14], micronutrient acquisition [8] and sulfur cycling [15]. Recent metagenomics and genomics data also indicate a role in carbohydrate degradation [14], including chitin [8]. However, studies performed so far were mostly based on the inspection of one or few *Endozoicomonadaceae* species, while family-wide, comparative surveys with multiple genera are lacking. Hence, this study inspected 42 *Endozoicomonadaceae* genomes (i.e., all genomes publicly available until May 2022), including cultured and uncultured lineages and all formally described genera, to determine the distribution and diversity of endo-chitinase-encoding genes and whether chitin hydrolysis is indeed a widespread capacity in this family. The full methodology employed in this study is documented in Additional file 1. Details on the genomes examined here and their annotations are available in Additional file 2.

## Results and discussion

**Fig. 1:**
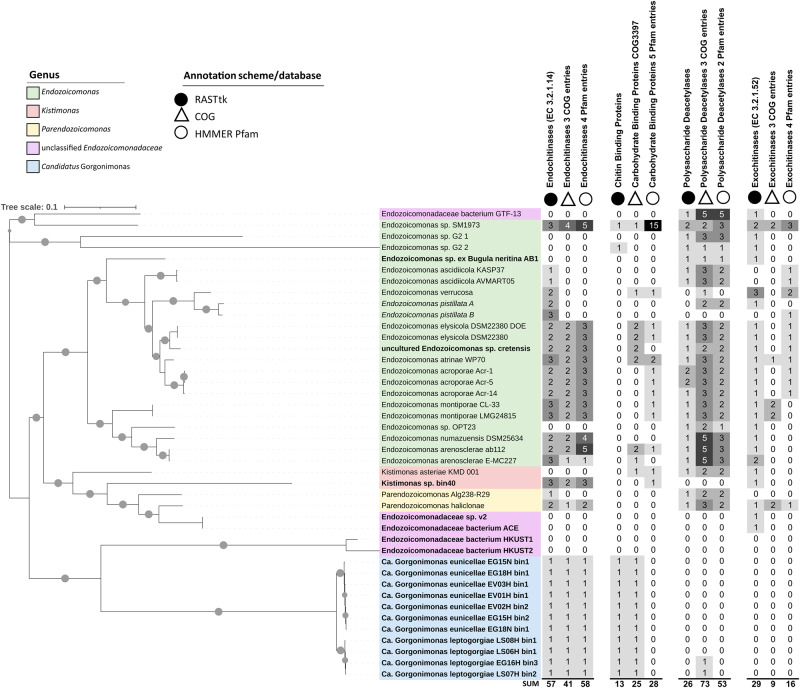
Chitin-degradation related genes present in the *Endozoicomonadaceae* family. The Maximum Likelihood phylogenomic tree of the *Endozoicomonadaceae* family on the left was constructed with the Insert Genomes into Species Tree—v.2.2.0 app of DOE Systems Biology Knowledgebase (KBase). This tree presents 42 genomes, including 19 metagenome-assembled genomes (MAGs, in bold), two single amplified genomes (SAGs, in italics), and 21 genomes from cultured representatives. Gray circles on the branches indicate bootstrap support of ≥75%. The tree is drawn to scale and was edited in iTOL. The columns next to the tree indicate the number of chitin-degradation related proteins/protein domains present on each genome. Chitin-degradation related genes were annotated using RASTtk v.2.0 (Rapid Annotation using Subsystem Technology toolkit; black circles), COG (Cluster of Orthologous Groups of proteins, empty triangles) and Pfam (protein families, empty circles) databases, respectively. Ca.—*Candidatus*. More information on the genomes used here, including their genome assembly accession numbers, is available in Table S1 of Additional File 2. Genome-wide COG and Pfam annotations of the 42 *Endozoicomonadaceae* genomes are available in Tables S3 and S4. Further details on the chitin degradation-related entries shown here can be found in Table S2 (RASTtk) and Table S5 (COGs, Pfams).

Genes coding for endo-chitinases (EC 3.2.1.14), the enzymes that cleave chitin polymers into oligomers [16], were found on 32 of 42 *Endozoicomonadaceae* genomes (Fig. 1 and Table S1), including representatives of all formally described genera and all *Candidatus* Gorgonimonas MAGs. Several genomes harbored more than one endo-chitinase encoding gene, resulting in 57 such genes detected across 32 genomes with a coding potential for chitin hydrolysis (RASTtk annotation, see also Table S2 for details). Of the 57 genes, 40 genes were found to code for glycoside hydrolase family GH18 endo-chitinases, the most common type of chitinase in bacteria [1]. In addition, we found four GH19-type endo-chitinases, one dual GH18-GH19 endo-chitinase and two GH6-type enzymes possibly indicating cellulase activity, while no GH domain was detected on the remaining 10 sequences. Twenty-nine *Endozoicomonadaceae* genomes also had genes coding for exo-chitinases/β-N-acetylglucosaminidases (EC 3.2.1.52, RASTtk annotation), which can cleave chito-oligomers to produce the mono-sugar N-acetylglucosamine [16]. Moreover, 26 genomes harbored polysaccharide deacetylases, indicating that *Endozoicomonadaceae* may also convert chitin into chitosan [1], and 13 had chitin-binding protein-encoding genes, revealing indeed a versatile chitin degradation machinery in this marine bacterial family.

Phylogenomic inference of the *Endozoicomonadaceae* family (Fig. 1) showed that strains without endo-chitinase encoding genes were usually not of the same species as those that carried endo-chitinases, indicating interspecific trait variation. Habitat origin, on the other hand, did not seem to be a determining factor, since both, genomes with and without endo-chitinases, derived from a variety of host animals and even non-host environments (Table S1). Of the 10 genomes that lacked endo-chitinase genes, five were MAGs and five were genomes from cultured representatives (Fig. 1). Likewise, genomes from cultured representatives with >99% genome completeness (e.g., *Endozoicomonas* sp. G2 1, *Endozoicomonadaceae* bacterium GTF-13, *Kistimonas asteriae* KMD 001) were found not to carry an endo-chitinase whereas multiple MAGs with 60–70% genome completeness did possess endo-chitinases (Table S1), indicating that neither cultivation status nor assembly completeness were determining factors for endo-chitinase presence across the surveyed genomes.

Nevertheless, the number of chitin-degradation related entries varied somewhat depending on the annotation tool/database used. For example, Clusters of Orthologous Groups of proteins (COG) and Protein families (Pfam) annotations identified 41 and 58 endo-chitinase domains (compared to 57 identified by RASTtk) on 26 out of 42 *Endozoicomonadaceae* genomes (Fig. 1, Tables S3–S5). The number of predicted exo-chitinases was lower with COG (*n* = 9) and Pfam (*n* = 16) annotations than with RASTtk (*n* = 29), while the number of chitin/carbohydrate binding domains (25 and 28) and polysaccharide deacetylases (73 and 53) was higher with COG and Pfam than with RASTtk (13 and 26). Quite congruent were the endo-chitinase predictions for the 11 *Candidatus* Gorgonimonas MAGs with all annotation schemes predicting one endo-chitinase per MAG (Fig. 1 and Table S1).

**Fig. 2:**
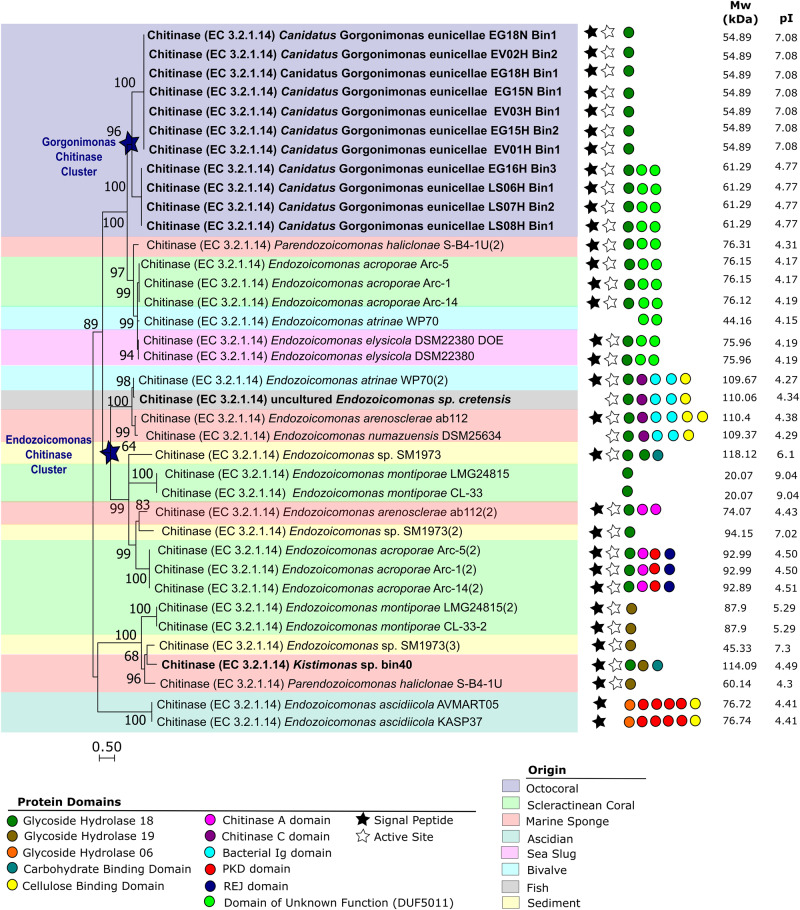
Phylogenetic analysis of endo-chitinases (EC 3.2.1.14) in the *Endozoicomonadaceae* family. The tree was produced using Mega11 with 37 endo-chitinase amino acid sequences, translated from full-length endo-chitinase gene sequences obtained from *Endozoicomonadaceae* genomes. Endo-chitinases from a MAG are highlighted in bold, the remaining endo-chitinase genes derived from cultured representatives. Sequences were aligned using MAFFT (Multiple Alignment using Fast Fourier Transform) v. 7.48. The evolutionary history was inferred using the Maximum Likelihood method based on a Whelan And Goldman (WAG) model. The tree with the highest log likelihood (−10396.47) is shown. A discrete Gamma distribution was used to model evolutionary rate differences among sites (5 categories (+*G*, parameter = 50.1306)). The rate variation model allowed for some sites to be evolutionarily invariable ([+*I*], 0.44% sites). The tree is drawn to scale, with branch lengths measured in the number of substitutions per site. There were a total of 340 amino acid positions in the final dataset. The tree was constructed with 1000 bootstrap repetitions, and values on the branches indicate bootstrap support of ≥60%. Blue stars on cluster nodes highlight genus-specific (*Candidatus* Gorgonimonas and *Endozoicomonas*, respectively) endo-chitinase clusters. Protein domains present on the endo-chitinase sequences are indicated by colored dots as identified in the legend below the tree. Black stars indicate the presence of a signal peptide and white stars the prediction of an active site. The theoretical average molecular weight (in kDa) and isoelectric point (pI) of each endo-chitinase (after removal of the corresponding signal peptide domain) is displayed on the right. The colored background shading indicates the origin of the *Endozoicomonadaceae* genomes as detailed in the legend below the tree. More information about the endo-chitinase sequences shown here is available in Table S6 of Additional file 2.

A phylogenetic analysis (Fig. 2 and Table S6) of 37 full-length, translated amino acid sequences showed that endo-chitinases of the 11 *Candidatus* Gorgonimonas MAGs formed a separate, well-supported cluster, unique of octocorals, and congruent with the distinct phylogenomic grouping of this candidate genus within the family (Fig. 1). Resembling species-level phylogeny, *Candidatus* Gorgonimonas endo-chitinases formed two subclusters (Fig. 2), indicating that each *Candidatus* Gorgonimonas species has its specific endo-chitinase. These endo-chitinases differed in amino acid (524 versus 577 aa) sequence lengths, predicted molecular weight (54.89 versus 61.29 kDa) and isoelectric point (7.08 versus 4.77), and by the presence of two Domains of Unknown Function (DUFA5011) in *Candidatus* Gorgonimonas leptogorgiae, which were absent in *Candidatus* Gorgonimonas eunicellae. Following a Blastp search, the closest endo-chitinases present in public databases were GH18 and DUF5011 domain-containing proteins from *Photobacterium* spp. (*Vibrionaceae, Gammaproteobacteria*). However, amino acid sequence similarity was low (~50%, see Table S6 for details), underlining the uniqueness of *Candidatus* Gorgonimonas endo-chitinases within the *Endozoicomonadaceae* family and *Gammaproteobacteria* in general.

Eighty-four percent of the *Endozoicomonadaceae* endo-chitinases depicted in Fig. 2 contained a signal peptide domain, responsible for transporting the enzyme outside of the cell [17] where endo-chitinases typically degrade the large, water-insoluble chitin fibers [1]. Moreover, 86% of these *Endozoicomonadaceae* endo-chitinases had an active site where the chitinous substrate is bound and the catalytic reaction initiated [18] (Fig. 2), suggesting that the endo-chitinases are functional. Several *Endozoicomonadaceae* genomes harbored two or three distinct endo-chitinases that differed significantly in their protein domains, molecular weight (from 20 kDa to 118 kDa) and isoelectric points (from 4.15 to 9.04) (Fig. 2). *Endozoicomonas montiporae*, *Endozoicomonas* sp. SM1973 and *Paraendozoicomonas haliclonae*, for example, harbored both GH18 and GH19-type chitinases. Possibly, these differences in protein sequence domains, molecular weight, and isoelectric points translate into distinct substrate affinities and/or enzymatic properties, such as different temperature and/or pH optima, or heavy metal tolerance, as recently observed in chitinases from *Vibrio* species [19]. Such chitinolytic plasticity could provide an advantage for *Endozoicomonadaceae* bacteria to cope with environmental changes in their natural habitat and with unique physical-chemical conditions and substrate quality and availability across different host-microbe symbioses.

We demonstrate that chitinase-encoding genes are widespread among *Endozoicomonadaceae* family members and frequently accompanied by auxiliary genes related to chitin breakdown, suggesting that these bacteria are well-adapted to process chitin-containing matrices in diverse marine micro-niches. *Endozoicomonadaceae* symbionts are thus likely important contributors to chitin turnover in filter- and suspension-feeding marine animals, capable of providing energetic, carbon and nitrogen-rich mono- and oligo-sugars to the holobiont. Given their ubiquity in the marine benthos, *Endozoicomonadaceae* bacteria could play key roles in carbon and nitrogen cycling on reefs and in marine ecosystems at large.

Studies of the kinetics and biochemical properties of endo-chitinases from the *Endozoicomonadaceae* family, including heterologously expressed endo-chitinases from uncultured organisms, such as *Candidatus* Gorgonimonas symbionts of octocorals, are needed to deepen our understanding of their physiology and function. This knowledge may open future opportunities for the circular blue bioeconomy sector since chitinases and chitin-derived products find various biotechnological and pharmaceutical applications [2, 3]. Moreover, future in-vivo studies using stable isotope probing will be helpful to determine the fate of chitin and to reveal chitin-associated cross-feeding mechanisms and food webs in coral and sponge holobionts. Such insights will contribute to a more holistic understanding of symbiont ecology and carbon and nitrogen fluxes in the marine realm.

### Supplementary information

Supplementary information.

Supplementary TableS1.

Supplementary TableS2.

Supplementary TableS3.

Supplementary TableS4.

Supplementary TableS5.

Supplementary TableS6.

## Data Availability

All genomes and MAGs used in this study are available in public databases, namely NCBI GenBank, or RAST, and genome assembly accession numbers are provided in Additional file 2 (Table S1).

## References

[1] Beier S, Bertilsson S (2013). Bacterial chitin degradation—mechanisms and ecophysiological strategies. Front Microbiol..

[2] Yadav M, Goswami P, Paritosh K, Kumar M, Pareek N, Vivekanand V (2019). Seafood waste: a source for preparation of commercially employable chitin/chitosan materials. Bioresour Bioprocess..

[3] Patel S, Goyal A (2017). Chitin and chitinase: role in pathogenicity, allergenicity and health. Int J Biol Macromol..

[4] Biancalana F, Kopprio G, Dutto MS, Berasategui AA, Fricke A, Garzón-Cardona JE (2017). Chitin determination on marine seston in a shallow temperate estuary (Argentina). Braz J Oceanogr..

[5] Zobell CE, Rittenberg SC (1938). The occurrence and characteristics of chitinoclastic bacteria in the sea. J Bacteriol..

[6] Raimundo I, Silva R, Meunier L, Valente S, Keller-Costa T, Costa R (2021). Functional metagenomics reveals differential chitin degradation and utilization features across free-living and host-associated marine microbiomes. Microbiome.

[7] Bartz J-O, Blom J, Busse H-J, Mvie JB, Hardt M, Schubert P (2018). *Parendozoicomonas haliclonae* gen. nov. sp. nov. isolated from a marine sponge of the genus *Haliclona* and description of the family *Endozoicomonadaceae* fam. nov. comprising the genera *Endozoicomonas*, *Parendozoicomonas*, and *Kistimonas*. Syst Appl Microbiol..

[8] Keller-Costa T, Kozma L, Silva SG, Toscan R, Gonçalves J, Lago-Lestón A (2022). Metagenomics-resolved genomics provides novel insights into chitin turnover, metabolic specialization, and niche partitioning in the octocoral microbiome. Microbiome..

[9] van de Water JAJM, Allemand D, Ferrier-Pagès C. Host-microbe interactions in octocoral holobionts—recent advances and perspectives. Microbiome. 2018;6:64. 10.1186/s40168-018-0431-6 PMC5880021 29609655

[10] Keller-Costa T, Lago-Leston A, Saraiva JP, Toscan R, Silva SG, Gonçalves J (2021). Metagenomic insights into the taxonomy, function and dysbiosis of prokaryotic communities in octocorals. Microbiome.

[11] Sweet M, Villela H, Keller-Costa T, Costa R, Romano S, Bourne DG (2021). Insights into the cultured bacterial fraction of corals. mSystems..

[12] Neave MJ, Michell CT, Apprill A, Voolstra CR (2017). *Endozoicomonas* genomes reveal functional adaptation and plasticity in bacterial strains symbiotically associated with diverse marine hosts. Sci Rep..

[13] Pogoreutz C, Oakley CA, Rädecker N, Cárdenas A, Perna G, Xiang N (2022). Coral holobiont cues prime *Endozoicomonas* for a symbiotic lifestyle. ISME J..

[14] Jensen S, Frank JA, Arntzen MØ, Duperron S, Vaaje-Kolstad G, Hovland M (2021). *Endozoicomonadaceae* symbiont in gills of *Acesta* clam encodes genes for essential nutrients and polysaccharide degradation. FEMS Microbiol Ecol..

[15] Tandon K, Lu C-Y, Chiang P-W, Wada N, Yang S-H, Chan Y-F (2020). Comparative genomics: dominant coral-bacterium *Endozoicomonas acroporae* metabolizes dimethylsulfoniopropionate (DMSP). ISME J..

[16] Cohen-Kupiec R, Chet I (1998). The molecular biology of chitin digestion. Curr Opin Biotechnol..

[17] Owji H, Nezafat N, Negahdaripour M, Hajiebrahimi A, Ghasemi Y (2018). A comprehensive review of signal peptides: structure, roles, and applications. Eur J Cell Biol..

[18] Liu S, Shao S, Li L, Cheng Z, Tian L, Gao P (2015). Substrate-binding specificity of chitinase and chitosanase as revealed by active-site architecture analysis. Carbohydr Res..

[19] He X, Yu M, Wu Y, Ran L, Liu W, Zhang XH (2020). Two highly similar chitinases from marine *Vibrio* species have different enzymatic properties. Mar Drugs..

