# Supplementary material for: Widespread occurrence of chitinase-encoding genes suggests the Endozoicomonadaceae family as a key player in chitin processing in the marine benthos
Source: ISME Commun. 2023 Oct 14;3:109. doi: 10.1038/s43705-023-00316-7 (PMC10576748; doi:10.1038/s43705-023-00316-7)
Supplement: Supplementary file 1 — Supplementary information. [file 43705_2023_316_MOESM1_ESM.docx]

**BRIEF COMMUNICATION - ADDITIONAL FILE 1 - METHODS**

**TITLE**

**Widespread occurrence of chitinase-encoding genes suggests the *Endozoicomonadaceae* family as a key player in chitin processing in the marine benthos**

**RUNNING TITLE**

**Chitinases in the *Endozoicomonadaceae* family**

**AUTHORS**

Daniela M. G. da Silva^1,2$^, Filipa R. Pedrosa^1$^, M. Ângela Taipa^1,2^, Rodrigo Costa^1,2^ and Tina Keller-Costa^1,2*^

**AFFILIATIONS**

^1^Department of Bioengineering, Instituto Superior Técnico, University of Lisbon, Av. Rovisco Pais, 1049-001 Lisbon, Portugal

^2^iBB - Institute for Bioengineering and Biosciences and i4HB - Institute for Health and Bioeconomy, Instituto Superior Técnico, Av. Rovisco Pais, 1049-001 Lisbon, Portugal

**^$^EQUAL CONTRIBUTIONS** these authors contributed equally to this work.

***CORRESPONDENCE** tinakellercosta@tecnico.ulisboa.pt, (+351) 21 841 3167

**METHODS**

Dataset

The 42 genome assemblies of cultured representatives (*n*=21), metagenome assembled genomes (MAGs; *n*=19) and single amplified genomes (SAGs; *n*=2) used in this study were downloaded in fasta format from public databases such as GenBank NCBI (1), IMG/M (Integrated Microbial Genomes and Microbiomes from DOE JGI) (2) and RAST (Rapid Annotation using Subsystem Technology) (3), corresponding to all *Endozoicomonadaceae* genomes publicly available on these platforms until May 2022. Genome assembly accession numbers, host species, and annotation details for chitin-degradation related genes are provided in Tables S1-S6 of Additional file 2.

Annotation of genes involved in chitin degradation

All genomes, MAGs and SAGs were uploaded to RAST (Rapid Annotation Using Subsystem Technology) v.2.0 (3) for genome-wide annotation with the RAST*tk* annotation scheme and to search for genes encoding endo-chitinases (EC 3.2.1.14), exo-chitinases (EC 3.2.1.52), polysaccharide deacetylases and chitin binding proteins. All endo-chitinase (EC 3.2.1.14) gene sequences were then downloaded from RAST in fasta format and converted into amino acid sequences using the EMBOSS Transeq sequence translation tool from EMBL-EBI (European Bioinformatics Institute) (4). The tool was used also to confirm gene completeness by verifying the presence of start and stop codons, respectively. Subsequently, annotation of protein family domains, signal peptides and active sites was performed on all endo-chitinase protein sequences using the HMMER scan tool from EMBL-EBI (4). Moreover, for signal peptide prediction, we also ran SignalP v. 6.0 (5) to consolidate the results from the HMMER scan tool which uses Phobius (6) to predict signal peptides. The theoretical, average molecular weight (MW in Dalton) and isoelectric point (pI) of each protein was calculated from full and signal-peptide deleted amino acid sequences using the tool Expasy (7) from SIB (Swiss Institute of Bioinformatics). MW and pI prediction of signal peptide deleted versions of the proteins was performed because signal peptides are usually cleaved off during transmembrane transport. The Protein Basic Local Alignment Search Tool Blastp (8) was used to search for closest sequence matches and identify chitinases present in public databases with similarity to the *Endozoicomonadaceae* endo-chitinases. In addition to the RAST*tk* annotation, genome-wide annotations based on COG (Clusters of Orthologous groups of proteins) and Pfam (Protein families) databases were performed with our in-house, automated genome annotation pipeline Melange as documented on GitHub (<https://github.com/sandragodinhosilva/melange>) and previously described (9, 10). The Pfam and COG outputs (Tables S3 and S4) were also screened for chitin-degradation related entries (see Table S5 for details), to identify features that were eventually missed by the RAST*tk* annotation.

Phylogenomic tree construction

To assess possible relationships between *Endozoicomonadaceae* phylogeny and the presence of chitin-degradation related genes (Fig. 1), a phylogenomic tree was constructed with the DOE Systems Biology Knowledgebase (KBase) (11) with all 42 publicly available *Endozoicomonadaceae* genomes, MAGs, and SAGs. Firstly, genome assemblies were imported to KBase in FASTA format and annotated with Prokka v1.14.5 (12). The phylogenomic tree was then constructed with the ‘SpeciesTreeBuilder’ v2.2.0 application, using the function “Insert Set of Genomes into Species Tree”. The ‘SpeciesTreeBuilder’ uses the FastTree2 (13) algorithm to infer Maximum-Likelihood phylogenies for large alignments. Alignments were based on a set of 49 core genes defined by Clusters of Orthologous Groups (COG) gene families. Graphical visualization and editing of the phylogenomic tree were done in iTOL v5 (Interactive Tree Of Life) (14). Thereafter, the number of proteins (or protein domains) per genome involved in chitin degradation (annotated with RAST*tk,* COG and Pfam databases) were respectively plotted next to the tree.

Phylogenetic analysis of endo-chitinases

To assess the phylogenetic relationships of the endo-chitinase (EC 3.2.1.14) sequences in the *Endozoicomonadaceae* family, a phylogenetic tree (Fig. 2) was constructed. Thirty-seven (of fifty-seven) full-length, endo-chitinase amino acid sequences from the *Endozoicomonadaceae* genomes were included in the phylogenetic inference. Sequences that were incomplete (i.e., start and/or stop codon missing) or very short (less than 150 aa in length) were excluded. Amino acid sequences were aligned using the MAFFT (Multiple Alignment using Fast Fourier Transform) v. 7.48 tool (15) from EMBL-EBI (4) with 1.53 gap opening penalty and 0.153 gap extension penalty (BLOSUM62 matrix). The alignment (fasta-format) was then imported into MEGA11 (16) to build the phylogenetic tree. The evolutionary history was inferred using the Maximum Likelihood method based on the Whelan And Goldman (WAG) model. A discrete Gamma distribution was used to model evolutionary rate differences among sites (5 categories (+*G*, parameter = 50.1306). The rate variation model allowed for some sites to be evolutionarily invariable ([+*I*], 0.44% sites). The WGA+G+I model was chosen as best-fitting model based on the ‘Find Best Protein Models’ test result from MEGA11. All positions with less than 85% site coverage were eliminated, i.e., fewer than 15% alignment gaps, missing data, and ambiguous bases were allowed at any position (‘partial deletion’ option). The phylogenetic tree was constructed using 1000 bootstrap repetitions. Style-editing of the tree was performed in Inkscape. Thereafter, the presence of protein domains, signal peptides, and active sites (annotated with HMMER Pfam as described above) on each endo-chitinase sequence was plotted next to the tree. The signal peptide-deleted, theoretical, average molecular weight (in kilodalton) and isoelectric point (pI) of each protein (calculated using Expasy (7) as described above) were also plotted next to the tree.

**REFERENCES**

1. Sayers EW, Bolton EE, Brister JR, Canese K, Chan J, Comeau DC, et al. Database resources of the national center for biotechnology information. Nucleic Acids Reseach. 2022;50(D1):D20-d6.

2. Chen IA, Chu K, Palaniappan K, Pillay M, Ratner A, Huang JH, et al. IMG/M v.5.0: an integrated data management and comparative analysis system for microbial genomes and microbiomes. Nucleic Acids Research. 2019;8(47(D1)):D666-D77.

3. Aziz RK, Bartels D, Best AA, DeJongh M, Disz T, Edwards RA, et al. The RAST Server: rapid annotations using subsystems technology. BMC Genomics 2008;9(75).

4. Madeira F, Pearce M, Tivey ARN, Basutkar P, Lee J, Edbali O, et al. Search and sequence analysis tools services from EMBL-EBI in 2022. Nucleic Acids Reseach. 2022;50(W1):W276-9.

5. Teufel F, Almagro Armenteros JJ, Johansen AR, Gíslason MH, Pihl SI, Tsirigos KD, et al. SignalP 6.0 predicts all five types of signal peptides using protein language models. Nature Biotechnology. 2022;40(7):1023-5.

6. Käll L, Krogh A, Sonnhammer ELL. A combined transmembrane topology and signal peptide prediction method. Journal of molecular biology. 2004;338(5):1027-36.

7. Duvaud S, Gabella C, Lisacek F, Stockinger H, Ioannidis V, Durinx C. Expasy, the Swiss Bioinformatics Resource Portal, as designed by its users. Nucleic Acids Research. 2021;49(W1):W216-W27.

8. Altschul SF, Gish W, Miller W, Myers EW, Lipman DJ. Basic local alignment search tool. Journal of Molecular Biology. 1990;215(3):403-10.

9. Keller-Costa T, Kozma L, Silva SG, Toscan R, Gonçalves J, Lago-Lestón A, et al. Metagenomics-resolved genomics provides novel insights into chitin turnover, metabolic specialization, and niche partitioning in the octocoral microbiome. Microbiome. 2022;10(1):151.

10. Silva SG, Paula P, da Silva JP, Mil-Homens D, Teixeira MC, Fialho AM, et al. Insights into the antimicrobial activities and metabolomes of *Aquimarina* (*Flavobacteriaceae, Bacteroidetes*) species from the rare marine biosphere. Marine Drugs. 2022;20(7):423.

11. Arkin AP, Cottingham RW, Henry CS, Harris NL, Stevens RL, Maslov S, et al. KBase: The United States Department of Energy Systems Biology Knowledgebase. Nature Biotechnology. 2018;36(7):566-9.

12. Seemann T. Prokka: rapid prokaryotic genome annotation. Bioinformatics. 2014;30(14):2068-9.

13. Price MN, Dehal PS, Arkin AP. FastTree 2 - approximately maximum-likelihood trees for large alignments. PloS One. 2010;5(3):e9490-e.

14. Letunic I, Bork P. Interactive Tree Of Life (iTOL) v4: recent updates and new developments. Nucleic Acids Research. 2019;47(W1):W256-W9.

15. Katoh K, Standley DM. MAFFT Multiple Sequence Alignment Software Version 7: Improvements in performance and usability. Molecular Biology and Evolution. 2013;30(4):772-80.

16. Tamura K, Stecher G, Kumar S. MEGA11: Molecular Evolutionary Genetics Analysis Version 11. Molecular Biology and Evolution. 2021;38(7):3022-7.
